# Comparison of two techniques for evaluation of pancreatic islet viability: flow cytometry and FDA/PI staining

**DOI:** 10.1186/1758-5996-7-S1-A247

**Published:** 2015-11-11

**Authors:** Natália Emerim Lemos, Cristine Dieter, Jakeline Rheinheimer, Bianca Marmontel de Souza, Rodrigo Carlessi, Cristiane Bauermann Leitão, Andrea Carla Bauer, Daisy Crispim

**Affiliations:** 1 Universidade Federal do Rio Grande do Sul (UFRGS), Porto Alegre, Brazil

## Background

Type 1 diabetes (T1D) accounts for approximately 10% of all diabetes cases, and it is caused by autoimmune destruction of pancreatic beta-cells, which leads to insulin deficiency and fates individuals to require insulin treatment to survive. Although important advances in the treatment of T1D have been achieved in recent yrs., this disease is associated with chronic complications that lead to high morbidity and mortality rates in young adults of productive age. In patients with unstable T1D, pancreatic islet transplantation is a therapeutic option to restore insulin secretion and improve glycemic control. However, the success of islet transplantation is dependent, in part, on the number of isolated islets as well as factors associated with their quality, which is assessed by functional and viability tests. In this context, the method currently used for islet viability evaluation [fluorescein diacetate (FDA)/propidium iodide (PI) staining] is not accurate enough, and new methods have been researched, such as flow cytometry.

## Objective

To compare two techniques used for islet viability evaluation: flow cytometry and FDA/PI staining.

## Methodology

Isolated islets of 10 male Wistar rats were used to evaluate cell viability. Upon FDA and PI staining, living cells convert the non-fluorescent FDA into the green compound fluorescein, while dead cells show red fluorescence in their nuclei due to penetration of PI through the permeabilized membrane. In a fluorescent microscope, 50 islets from each animal were analyzed by two researches, and the percentages of viable and dead cells per islet were estimated. For flow cytometry islets were disperse, and single cells were incubated with 7AAD fluorophore (which dyes necrotic/late apoptotic cells) and Annexin V-FITC monoclonal antibody (which identifies early apoptotic cells). One thousand cells were evaluated by the cytometer.

## Results

The Pearson correlation between the two techniques was 0.6 (P=0.047), indicating a moderate correlation. The mean viability evaluated using flow cytometry was slightly higher than the mean viability estimated using FDA/PI staining (95.5±1.4% vs. 89.5±5.0%, respectively; P=0.002).

## Conclusions

Although flow cytometry is more expensive and time-consuming for evaluation of viability than FDA/PI, it is a quantitative technique, not dependent of the researcher eye. Thus, flow cytometry should be the technique of choice for more effective determination of islet viability.

